# COVID‐19‐induced adult multisystem inflammatory syndrome and fatal acute limb ischaemia

**DOI:** 10.1002/rcr2.886

**Published:** 2021-12-01

**Authors:** Hiroshi Kobe, Akihiro Ito, Tadashi Ishida

**Affiliations:** ^1^ Department of Respiratory Medicine Ohara Healthcare Foundation, Kurashiki Central Hospital Okayama Japan

**Keywords:** acute limb ischaemia, adult multisystem inflammatory syndrome, COVID‐19

## Abstract

A case of coronavirus disease 2019 (COVID‐19)‐induced adult multisystem inflammatory syndrome (MIS) and fatal acute limb ischaemia is presented. Arterial thrombosis and MIS are reported as complications of COVID‐19. This case further highlights that arterial thrombosis and MIS can occur in COVID‐19.

## Graphical abstract

A case of coronavirus disease 2019 (COVID‐19)‐induced adult multisystem inflammatory syndrome and fatal acute limb ischaemia is presented.

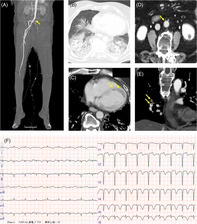

## CLINICAL IMAGE

**FIGURE 1:**
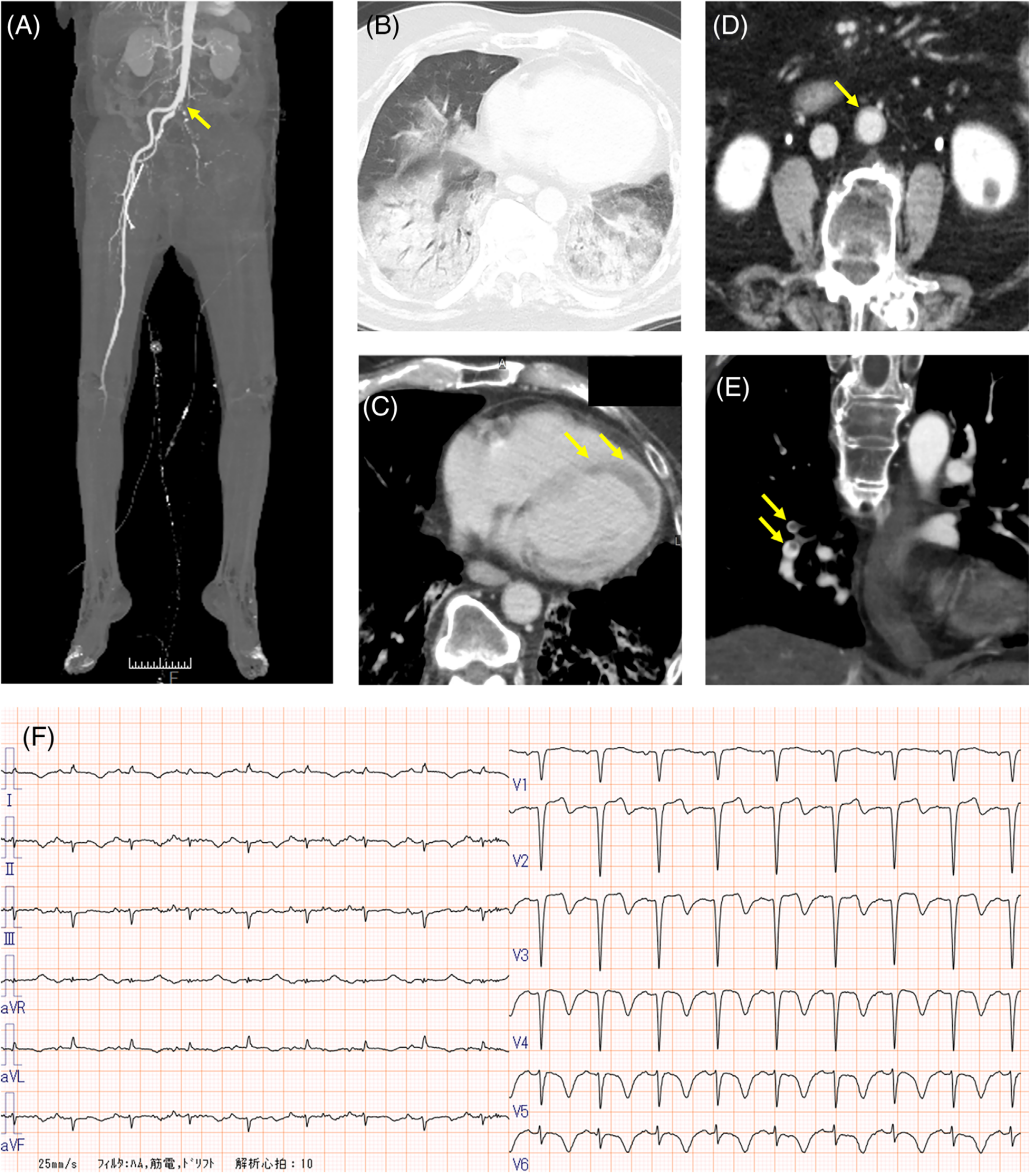
(A) Three‐dimensional computed tomography (CT) angiography. Complete occlusion at the branch from the abdominal aorta to the left common iliac artery (arrow). (B) Chest CT shows bilateral lung diffuse consolidation and ground‐glass opacity. (C) Contrast‐enhanced CT confirms contrast‐delayed areas (arrows) in the myocardium surrounding the left ventricle with suspected endocarditis or myocarditis. (D) Contrast‐enhanced CT confirms the aortic wall's contrast effect (arrows) with suspected aortitis. (E) Contrast‐enhanced CT confirms multiple pulmonary emboli in the right pulmonary arteries (arrows). (F) Electrocardiogram shows ST elevation and inverted T waves in the precordial leads

**FIGURE 2:**
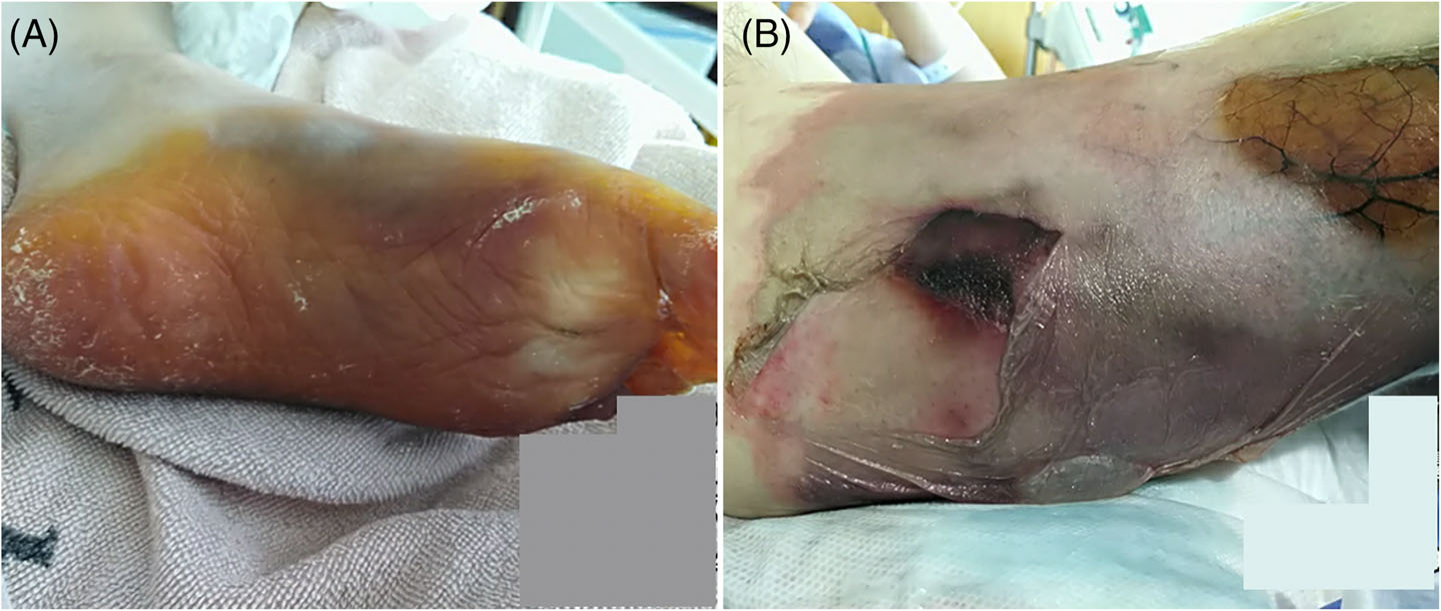
(A) Ischaemia seen and colour worsens on the patient's left sole. (B) Blisters seen on the surface of the patient's left medial thigh worsen over time

A 72‐year‐old man presented to our hospital with a 1‐week history of dyspnoea and pain in the left lower leg. The patient had a history of untreated type 2 diabetes mellitus (HbA1c 14.1%; reference range, 4.6%–6.2%) and no history of smoking. No heart disease or peripheral vascular disease was found previously. Laboratory examinations showed elevated levels of d‐dimer (258.8 μg/ml; reference range, 0.0–1.0 μg/ml) and C‐reactive protein (31.57 mg/dl; reference range, 0.0–0.14 mg/dL), and decreased platelet count (83,000/μl). A nasopharyngeal swab was positive for coronavirus disease 2019 (COVID‐19) on real‐time polymerase chain reaction testing. Computed tomography showed lower extremity arterial thrombosis, COVID‐19 pneumonia, myocarditis, aortitis and pulmonary thromboembolism (Figure 1A–E). The electrocardiogram showed ST elevation and inverted T waves in the precordial leads (Figure 1F). Cyanosis and multiple blisters occurred after hospitalization (Figure 2). During the placement of a central venous femoral vein catheter, the femoral artery was accidentally punctured; thus, anticoagulants were not administered due to bleeding risk. Open surgical or endovascular thromboembolectomy was not performed due to the patient's poor general condition. COVID‐19‐induced adult multisystem inflammatory syndrome (MIS)^1^ and acute limb ischaemia were diagnosed. The patient died on the 17th day of hospitalization due to uncontrolled circulatory failure. According to the criteria of the International Society on Thrombosis and Haemostasis,^2^ the patient's total score for disseminated intravascular coagulation was 6 points, indicating the presence of overt disseminated intravascular coagulation. COVID‐19 patients may present with symptoms of thrombosis or MIS, and appropriate testing is necessary.

## CONFLICT OF INTEREST

None declared.

## AUTHOR CONTRIBUTION

Hiroshi Kobe: writing original draft. Akihiro Ito: supervision, writing, review and editing. Tadashi Ishida: visualization, writing, review and editing.

## ETHICS STATEMENT

The authors declare that appropriate written informed consent was obtained for the publication of this case report and accompanying images.

## Data Availability

Data sharing is not applicable to this article as no new data were created or analyzed in this study.
